# Publication bias *in situ*

**DOI:** 10.1186/1471-2288-4-20

**Published:** 2004-08-05

**Authors:** Carl V Phillips

**Affiliations:** 1 Center for Philosophy, Health, and Policy Sciences, Inc., Houston, USA; 2 Management, Policy and Community Health Division, University of Texas School of Public Health, RAS E-311, 1200 Pressler, Houston, TX 77225, USA; 3 Center for Clinical Research and Evidence-Based Medicine, University of Texas Medical School, Houston, USA

## Abstract

**Background:**

Publication bias, as typically defined, refers to the decreased likelihood of studies' results being published when they are near the null, not statistically significant, or otherwise "less interesting." But choices about how to analyze the data and which results to report create a publication bias within the published results, a bias I label "publication bias *in situ*" (PBIS).

**Discussion:**

PBIS may create much greater bias in the literature than traditionally defined publication bias (the failure to publish any result from a study). The causes of PBIS are well known, consisting of various decisions about reporting that are influenced by the data. But its impact is not generally appreciated, and very little attention is devoted to it. What attention there is consists largely of rules for statistical analysis that are impractical and do not actually reduce the bias in reported estimates. PBIS cannot be reduced by statistical tools because it is not fundamentally a problem of statistics, but rather of non-statistical choices and plain language interpretations. PBIS should be recognized as a phenomenon worthy of study – it is extremely common and probably has a huge impact on results reported in the literature – and there should be greater systematic efforts to identify and reduce it. The paper presents examples, including results of a recent HIV vaccine trial, that show how easily PBIS can have a large impact on reported results, as well as how there can be no simple answer to it.

**Summary:**

PBIS is a major problem, worthy of substantially more attention than it receives. There are ways to reduce the bias, but they are very seldom employed because they are largely unrecognized.

## Background

A study is more likely to appear in the literature, and thus be indexed and publicly available, if it shows a strong, statistically significant, or otherwise "better" result. A study that does not show such results has a greater chance of remaining hidden in a file drawer, either because the author (or funder) does not think the result is worth mentioning, or because journals are less interested in publishing studies that find "nothing". This form of publication bias, wherein readers have access to only a biased sample of the full distribution of results, is well studied, particularly in the literature on meta-analysis and other systematic reviews where the problem is most apparent (a primer on the topic can be found at the Cochrane Collaboration webpage [1]). Even well-read lay people are, at the time of this publication, aware of the problem due to the controversy about pharmaceutical companies selectively releasing research about their products.

Far less attention is paid to the bias that occurs when some results from a study are published, but the choice of *which *results to publish produces bias. As with traditional publication bias, the tendency is to analyze data and choose to present results which are statistically significant, further from the null, or closer to what the researchers believe is the true value. The implications for the literature as a whole, though, are much the same as the file drawer bias. I label this "publication bias *in situ*" (PBIS) because the biased reporting of study findings exists within each individual research report (with any metaphoric references to cancer – the usual context of the phrase "*in situ*" in health science –  left as an exercise for the reader). Despite the similarities between the file drawer bias and PBIS, there are fundamental differences. In particular, the bias from some studies having no published findings exists only at the level of the whole literature (no particular study can be said to be biased), while PBIS exists within the results reported from a single study (and thus exists in the literature as a whole by aggregation). More practically, PBIS is substantially more difficult to even identify, let alone correct.

This paper defines and describes PBIS and identifies some of the choices that create it. The purpose of the paper is not to make novel technical or statistical claims. Indeed, there is probably no single statistical observation here that will not be clear to a skilled data analyst (or, indeed, that could not be explained to anyone competent in middle-school-level math). The literature includes many observations about issues relating to PBIS. Yet the challenge to the validity of the entire health science literature posed by PBIS – arguably a greater issue than conflict of interest, traditional publication bias, or any other commonly discussed threat to the integrity of the literature – has not received the attention it deserves.

In the literature about meta-analysis and systematic reviews there is substantial attention to file-drawer publication bias (though most of this considers only bias from reporting statistically significant results, ignoring preferences for reporting more dramatic results or those that agree with authors' or journals' previous reports). But there is considerably less attention to possible picking and choosing which study results to report or statistical methods to use. To the extent that this is considered, it is often bundled with questions about whether *ex ante *protocols were stated and followed, and is thus put on par with many protocol violations that could be considered mere technicalities. For example, a few years ago, a highly-publicized meta-analysis [2] called into question the evidence on the benefits of screening mammography by citing apparent problems in the methods of most of the relevant randomized trials. But little was done to distinguish a few of these problems that appeared to be PBIS and others that were minor technical points.

The literature and pedagogy related to observational inference, where the potential for PBIS is considerably greater than for well-designed experiments, seems to pay even less attention. Epidemiology textbooks typically discuss some of the methods that can reduce PBIS (e.g., well-defined protocols), but say little or nothing about the possible bias. Indeed, many study and data-analysis methodologies (some of which are noted below) that are typically taught in classes or by apprenticeship seem designed to create PBIS. Despite this, highly-trained experts summarize claims reported in the literature without mention of the likely bias, indicating an unawareness of the major implications of PBIS.

In a notable exception, Hahn and colleagues [3,4] discussed some sources of PBIS and argued that they receive too little attention compared to bias from selective publication. Their analyses addressed selective reporting of subgroup results in the context of randomized trials, a topic further discussed below, and reporting a selected subset of multiple measured endpoints. They do not mention the other sources of PBIS discussed below. Unfortunately, their findings do not appear to have had the impact they deserved.

It is not clear how common PBIS is or how large the resulting bias, but a few efforts to find it suggest that the potential is great enough that it deserves much more attention. (Labeling might matter: Hahn et al. label the problem "within-study selective reporting." The more dramatic name suggested here, with its emphasis on the bias that results, might catch more attention.) Only by systematically addressing the problem are we likely to substantially affect it. Moreover, as will be discussed, statistical and research methods that ostensibly address some of the sources of PBIS are unsatisfactory, and a systematic attempt should be made to find better solutions.

## Discussion

### Many degrees of freedom

Reviewers (systematic or otherwise) of the literature can only see what researchers choose to report and highlight in their publications, and that choice can be biased in a number of ways. Researchers have great freedom in deciding exactly what to analyze, how to analyze it, and what to report. All research results are derived from data that can be used to measure many associations. Even the most narrowly focused clinical trial can be analyzed with the endpoint defined in different ways, stratified by age, etc. As with traditionally defined publication bias, the analyses that are deemed unworthy of publication are largely invisible to the scientific and policy community.

Among the dimensions of freedom researchers have in deciding what to analyze and report are three choices illustrated by examples in this paper:

(1) Which exposures and outcomes to consider in datasets with many variables.

(2) Which functional forms to use to represent variables (e.g., how to divide continuous variables into categories).

(3) Whether to conduct separate analyses by subgroup, and which subgroup results to emphasize.

Making such choices is a legitimate part of research. Indeed, the choices must be made. But when those choices are primarily driven by what produces stronger (or otherwise "better") results, bias is created. This creates a difficult challenge: It is easy to recognize traditional publication bias (paper in journal = no contribution to bias; paper in file cabinet = contribution to bias). But since there is no clearly correct option for any of the above choices (indeed, any particular analysis gives the right answer to *some *question), there is no clearly wrong choice, and thus no clear way of concluding that a particular choice was biased. Fortunately, as should become apparent from the following analysis, PBIS results less from the choices made and more from what the choices are based on (which can often be inferred) and, to an even greater extent, the generally overlooked issue of how the results are presented (which can be easily observed).

Publication bias, either PBIS or the file-drawer effect, can be seen most clearly as an interaction between random errors and researcher choices (e.g., when random sampling error leads to a weaker result, the result is less likely to be reported), creating a bias from what would be unbiased random variation. Systematic errors (confounding, measurement error, etc.) and methods for correcting for them create many additional opportunities for PBIS; however, for simplicity, systematic errors are ignored in this paper.

### Multiple analyses from the same data

Choice (1) in the above list has probably received the most attention in previous literature (for example, debates over whether to correct for multiple hypothesis testing and the appropriateness of data dredging). Despite the disproportionate attention, this choice is probably not the major source of PBIS, but it provides a familiar starting point.

Many epidemiologic datasets are characterized by thousands or even millions of possible combinations of exposures, endpoints, and covariates. It is frequently assumed that statistical science tells us the "right" way to deal with this challenge, but current practice (not to mention the confusion of students coming out of epidemiology and biostatistics classes) makes clear that there is substantial disagreement among viewpoints about how to apply statistical methods when dealing with multiple hypotheses or measurements. Further consideration makes it clear that statistical rules cannot actually provide clear answers.

At one extreme are viewpoints such as, "We must correct measures of statistical certainty (significance levels for p-values (α-levels) or confidence interval widths) whenever multiple comparisons are made using the same dataset" and even, "statistical analysis can only be legitimate for a short list of pre-specified hypotheses." At the other extreme are viewpoints such as, "regardless of how many comparisons are examined, each can be considered and statistically tested as if it were the only one," and, "it does not matter at all if a hypothesis was proposed after looking at the data." The impasse in this debate seems to stem from both sides attacking straw men, without recognizing that each side has a stronger case some of the time. This can be illustrated with examples.

#### Example: unrelated results from the same dataset

A cohort dataset originally used to report the relationship of drinking water source and the occurrence of *Helicobactor pylori *infection contains data that is later used to look at the relationship of household crowding and performance in school. It is difficult to understand why we would make an adjustment when doing the second analysis because we have already done the first (or, worse, disallow the analysis because it was not pre-specified in the study or because we have already "used up" our .05 worth of α with the *H. pylori *analysis, and so cannot analyze anything more with this data at all). A logical extension of that argument would be to consider the dataset that contains all quantitative human knowledge (which is logically an epistemologically legitimate definition), and declare that we have to adjust for every statistical analysis ever done, effectively precluding further statistical analysis.

#### Example: multiple comparisons that will support claims of the "same" relationship

Researchers investigate the hypothesis that poor nutrition increases the risk of *H. pylori *infection. The dataset contains dozens of different measures of food and nutrient intake, as is usually the case for nutrition data. This, plus multiple diagnostic tests for *H. pylori *which are sometimes discordant, creates a large number of statistical comparisons, any of which could be described as supporting the plain language claim, "poor nutrition affects *H. pylori *status." A typical approach is to find individual comparisons that support the hypothesis, presenting only these comparisons with statistical tests as if each were the only analysis conducted. The claim about the relationship between the particular measure of nutritional status and the particular measure of *H. pylori *status is accurate, as are the test statistics reported for that association. But the plain language conclusion (which would probably be drawn) was very likely to be supported by some relationship in the data by chance alone, even in the absence of any true underlying association. This fact is obscured by the reported unadjusted tests statistics or confidence intervals.

As the second example illustrates, unrestricted picking and choosing of comparisons leads to publication bias. A lot of associations that were not deemed worthy of reporting never appeared in the literature, while the few that were "interesting" did. This problem is well known (though few probably realize that it can lead to hundreds of instances of publication bias, *in situ *within a single published article, making it a bias of much greater magnitude than the file-drawer effect).

The solutions offered by statistical rules – corrections for multiple hypothesis testing or restricting analysis to *ex ante *hypotheses – is inadequate. Such rules produce absurd implications, noted in the first example. Trying to eliminate the absurdity by exempting from statistical adjustment analyses with disjoint exposures and outcomes, as in the first example, does not work; the second example offers options for disjoint analyses also. Most important (and widely overlooked), correcting for multiple comparisons does not affect the reported biased estimates of effect size; changing test statistics and confidence interval widths *does nothing to reduce the bias*. This alone shows that the standard statistical corrections for multiple tests do nothing to solve the problem. The other standard method for trying to reduce PBIS, rules that limit analyses to pre-specified hypotheses and protocols, will throw away a lot of potentially valuable findings and is nearly impossible to operationalize because detailed protocols require advanced knowledge that may not exist and can never be specified unambiguously.

Frequentist statistical theory cannot offer a solution to this problem because PBIS, like the file-drawer problem, is not a matter of statistics. The second example illustrates where the problem primarily lies: in the plain-language reporting of results. The statistics that describe the relationship between a particular exposure and outcome measure could be exactly right, but the claim about good nutrition (as a generic concept) and infection status (as if we had a gold standard measure), which will likely be emphasized in the paper and its title (and press releases) and will likely stick readers' minds, might be misleading. Consider how the result would be interpreted if there were a table reporting every analyzed comparison, most of which showed little or no association. Most scientifically literate readers would realize the result was not so convincing, even though those same readers seldom think to object when – as is typical – only one or a few results are reported. By contrast, if the researchers in the first example reported the result of the previous study, it would be unlikely to change most readers' assessments.

This suggests the simplest partial solution to the problem. By reporting a table of results from other comparisons considered, researchers could report their interesting result (rather than not informing the world due to the lack of a specific *ex ante *hypothesis or having "used up" the α), but without creating the PBIS that would result otherwise. Indeed, this appears to summarize the most obvious generic rule to reduce PBIS (and publication bias in general): publish everything.

An immediate implication of this is that online publications, like this journal, allow researchers to publish less biased articles. Online articles can usually be whatever length is appropriate to report the results (which is of particular value in the health sciences, where paper journals have extremely restrictive length limits), and can include dozens or thousands of alternative analyses in appendices or links to data or software that allow the reader to review still more results. Of course, this opportunity is beneficial only if authors choose to take advantage of it (or editors and reviewers demand that they do).

### Bias from the choice of functional form

The implications of choice (2), the functional form for variables, can be clearly illustrated with a simple example. Consider a study with an exposure variable measured as 10 ordered categories (i.e., values 1,2,...,10, with larger numbers representing greater exposure). Assume researchers wish to analyze the association of a disease endpoint and a dichotomous definition of exposure. If there is no clear cutpoint for defining exposure, there are many options. There are 9 cutpoints that divide the observations into two categories, defining those above the cutpoint to be exposed and those below unexposed. Other options include comparing a group of highest categories to a group of lowest categories, leaving out the middle, such as 8–10 versus 1–3, yielding an additional 36 possibilities.

How will the researchers choose a definition of exposure? A typical procedure is to let the data inform the choice: The cutpoint that provides the clearest contrast between the exposed and unexposed is judged to be the right one, the "most sensitive" to the presumed effect. It should be immediately obvious that this procedure will bias the result away from the null.

To illustrate this, consider a case-control study with 200 subjects (throughout the examples, subjects are half cases and half non-cases). Calculations for this example and others are based on Monte Carlo simulation of different realizations of the data based on the assumed underlying relationship. All simulations were performed using Crystal Ball (Decisioneering Inc., Denver, Colorado, USA).

Assume that each of the 10 exposure categories is equally likely for cases and non-cases. If the researchers consider only the 9 cutpoints that dichotomize the data and choose the cutpoint for "exposed" to get the largest odds ratio (OR), the median result will be 1.5. Since the exposure and disease are not associated, this is clearly an upward bias. For those inclined to focus on statistical significance, the chance of observing a significant positive association at a one-tailed significance level of .025 is 13%. (Of course, for any single definition of exposure, the median OR is 1.0 and the chance of seeing a significant relationship is about 2.5%.)

Even if researchers do not analyze their data in every possible way and report the strongest association, any decision to report results that is based on associations in the data (e.g., choosing between a cutpoint at 5 or at 6 based on which produces a stronger association) will create bias. Although this observation should be obvious to anyone with an understanding of statistics, letting the data have some influence on the choice is probably more the rule than the exception among researchers. It is often defended on the grounds that there was no way to know what the "right" cutpoint was before doing the study, and the study data is the only existing answer to the question. This is a legitimate point, but it does not reduce the resulting bias. Less scrupulous researchers – who are trying to support a preferred answer to further a policy agenda or advance their careers – need make no such explanation and can intentionally choose the extreme results.

As with the file-drawer effect, results in the literature will tend to show effects greater than the true value. Most important, whichever analysis is reported, the plain-language result will be "we found an association between the exposure and the disease," and so collections of studies that each report an exposure-disease comparison with a greater-than-average association, and will seem to be confirming the same result, even though the comparisons are not the same.

Extending this example to illustrate how PBIS can compare to traditional publication bias, assume now that there is a positive association between the exposure and disease. In particular, non-cases are still equally likely to be in each of the ten categories, while cases have respective probabilities for each category of (.069, .072, .077, .084, .093, .102, .112, .121, .131, and .139). These values were chosen so that the true OR is similar, whichever of the 9 cutpoints is chosen (for those interested, the numbers follow a logistic curve). True ORs round to 1.5 for all cutpoints.

Consider a collection of studies of varying sizes, with fewer larger studies, as we would typically see in the literature, specifically 100 randomly generated studies (more than would likely exist, but better to illustrate) of random size (drawn from a triangular distribution with modal probability at a minimum value of 100 subjects, diminishing linearly to a maximum of 1600 subjects). Note that to avoid committing the very type of transgression discussed in this paper – repeating an analysis until "good" results are found – the reported results are from the first and only run of the simulation.

**Figure 1:**
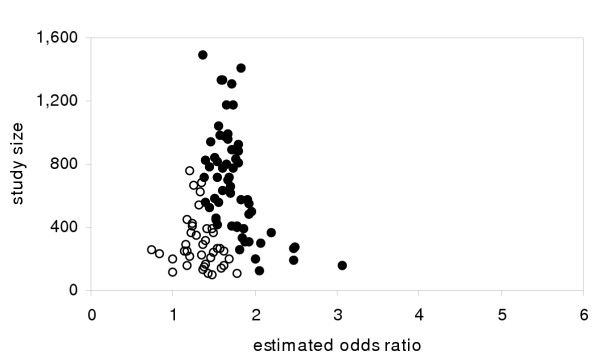
Traditional publication bias from simulated studies. Simulated results from case control studies with varying populations (half cases, half non-cases) for true odds ratio of 1.5. Solid circles represent statistically significant results at the 2-tailed, .05 level. (Note: x-axis scale chosen for compatibility with other figures.)

For a single definition of "exposed" (values >5), a typical result appears in Figure 1 in the form of a funnel plot of study results vs. study size [1,5,6]. The results that are statistically significantly different from 1.0 at the two-tailed .05 level are represented by solid dots. The other results (represented by open circles) might never be reported – the simplest form of publication bias – though they could be inferred from the asymmetry of the distribution that would occur if only the significant results were published. Notice that the distribution for all the studies is unbiased and would lead to an estimate very close to 1.5, while a naive summary estimate based only on the statistically significant studies (possibly the only ones published) could almost double the estimated effect size.

**Figure 2:**
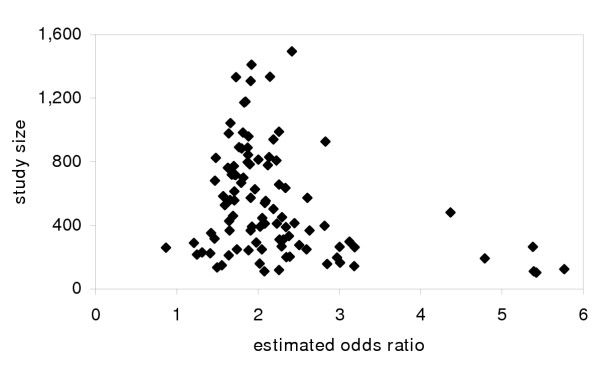
Publication bias *in situ *from simulated studies. Simulated results from case control studies with varying populations (half cases, half non-cases) for true odds ratio of 1.5. For each simulated study, the largest odds ratio (choosing among 9 different cutpoints for exposure definition) is reported. (Note: one outlier odds ratio estimate not shown.)

Compare this to the results for the same 100 studies where the cutpoint is chosen based on the largest OR (Figure 2). The distribution is also substantially biased, with PBIS leading to results above the single-definition results of 1.5. A summary estimate of effect size would turn out to have substantially greater bias than would reporting only significant results as in Figure 1. Notice that though there is a skew, it is much harder to discern a pattern like the asymmetry in Figure 1 that would show a systematic reviewer that the literature is biased.

Unbiased random errors, when combined with picking and choosing functional forms, lead to biases in reported results. A solution to this problem is much less obvious than its existence.

The commonly proposed solution of only reporting results for pre-specified functional forms is not satisfactory, because it is difficult to enforce (most every pre-specification has some room for interpretation in retrospect; intentional cheating is difficult to detect; there may be little basis for selecting any particular pre-specified functional form) and it forces us to ignore real unpredicted findings. Sticking to pre-specified analyses is especially unrealistic in research studies that collect data on many different risk factors and outcomes. Simply labeling all results that were not pre-specified with the caveat, "hypothesis generating," accomplishes nothing. If such results were actually treated as not yet "real", the problems of determining exactly which results were pre-specified and the loss of important serendipitous findings are reintroduced. Of course, results with the caveat are very seldom treated as less real than any others in the literature. Moreover, the "generated" hypotheses will never be retested in exactly the form reported, so the label is simply disingenuous. In sum, the proposed solutions to this type of PBIS are no more realistic or satisfactory as solutions than trying to eliminate traditional publication bias by requiring that all studies be adequately powered.

**Figure 3:**
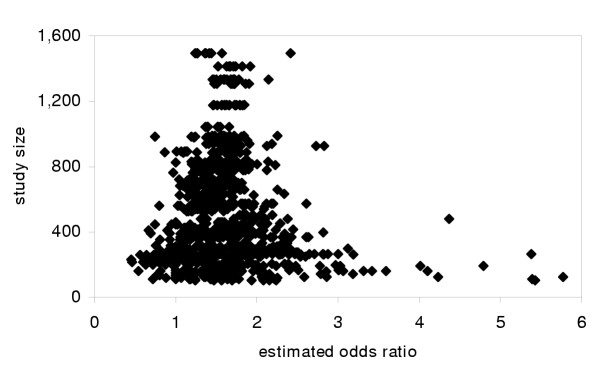
Publication bias *in situ *eliminated by reporting all results. Results from Figure 2, but with results for all possible cutpoints reported.

A better family of solutions would be to establish a standard of reporting results for alternative variable definitions (perhaps in online appendices). Not only does this directly reduce PBIS by publishing more results, but it provides readers with a choice of results if they prefer different definitions (information that would be discarded by a pre-specified hypothesis rule). If results for every cutpoint from the 100 trials in the example were reported, the results, as pictured in Figure 3, would be unbiased. Naturally, researchers could emphasize the variable definitions they think best, but by acknowledging other possibilities they would be forced to justify their choice.

In many cases there will not be an obvious short list of variable definitions, but some alternative definitions should be obvious and others could be found in previous literature. A simple, but very useful, improvement would be a standard practice of reporting the closest possible replication of previous published analyses using data from the new study. This would directly address the problem of PBIS resulting from data-driven picking and choosing of functional forms (though it might require the cooperation of previous authors to provide details about what analysis they reported, given the typically abbreviated reporting of methods in published papers – another problem with paltry word limits). Repeating whatever analyses that previous researchers happened to choose is somewhat arbitrary, but each round of new research can also add a new preferred functional form. The key is that results based on previously published functional forms cannot be data driven and, unlike the standard practice of new studies that make different comparisons but describe them with the same plain language, would actually replicate (or fail to replicate) previous results.

### Bias from the analysis of subgroups

In 2003, VaxGen Corporation (Brisbane, California, USA) released results of a large HIV vaccine trial in the United States, one of the highest-profile clinical trials of the year. The disappointing result showed a trivial reduction in incidence among the treatment group compared to the placebo group. But the three non-white racial groups (black, Asian, and "other") each showed a substantial reduction in incidence (Table 1). VaxGen reported the overall failure of the trial to the popular and business press [7,8]. A technical report describing the drug and the trial results, written by a third party, appeared later in an indexed journal [9], though the *New York Times *articles actually contained more complete study results. VaxGen tried to salvage some hope for the drug by pointing out the results for non-whites, suggesting that maybe it held promise for some populations [8]. VaxGen's search for a silver lining resulted in rash of criticism from the research community (focused on the reporting of a result that was not a pre-specified hypothesis and the failure to correct for multiple hypothesis testing) and a shareholder lawsuit, alleging that statistically illicit reporting was used to inflate stock prices [9-12].

**Table 1:** Results of VaxGen HIV vaccine trial

	total	vaccine		placebo		RR
			
	subjects	subjects	incidence	subjects	incidence	
white	4511	3003	179	1508	81	1.11
nonwhite	498	327	12	171	17	0.37
black	314	203	4	111	9	0.24
Asian	73	53	2	20	2	0.38
other	111	71	6	40	6	0.56
total	5009	3330	191	1679	98	0.98

Source: The *New York Times *[7] and author's calculations.

Given the failure to publish the results in a scientific journal, some might argue that VaxGen was guilty of traditional publication bias – not publishing unfavorable results about its products – a charge that is currently being leveled at many drug companies. However, the company actively released fairly complete study results and accompanying analysis to the press, and the results appeared in forums that are more widely read than all scientific journals combined, so the results were clearly not buried in a file drawer.

But despite reporting their results, VaxGen was biasing what they published. Singling out of the result for non-whites is a clear case of PBIS. VaxGen did not give equal emphasis to the apparently harmful effect of the vaccine for whites. The company's subsequent report that the different results for whites and non-whites could not be due to chance [9] does not diminish the apparent bias, since it basically repeats the same information contained in the original (data-driven, biased) reporting of the result for the non-white subgroup.

Setting aside some controversy that erupted about systematic errors in the data, what can we say about the results from the perspective of PBIS and chance alone? To look at the result for non-whites, with its one-tailed p-value of .002, ignoring the fact that the subgroup definition was clearly data-driven, would overstate the finding, as suggested in the preceding examples. But a naive correction for multiple hypothesis testing would make the opposite error. Setting aside the possibility that other covariates would have been used to stratify the data had they produced subgroups with positive findings, the combination of the 4 racial groups implies a test of 2^4^-1 = 15 different hypotheses. To adjust for 15 implicit hypotheses makes it very unlikely that any will pass the statistical test, including the population as a whole, even for substantial associations.

An alternative is to ask the question, "if the vaccine has no effect, what are the chances of seeing, in any racial group or combination thereof, a result at least as strong as the observed 63% reduction?". Phrased that broadly, simulation shows the answer is about 20%. However, most of the 20% comes from the unstable results for the two smallest groups, Asians and "other". Restricting the analysis to combinations of racial groups that contain black, with or without Asians or other, the probability is only 2.1% (the probability of seeing a 63% reduction by chance alone for any group that includes the whites is vanishingly small).

So, what is the right answer? We must return to the observation that there is never a single Right Answer from a study; the quality of an answer always depends on what question was being asked. Did VaxGen find a successful vaccine? Clearly not, as the relative risk for the whole population shows. Should the result for non-whites be considered unlikely to be due to chance (i.e., statistically significant)? It depends on whether you consider it the answer to the question "does the vaccine show a result for non-whites?", in which case the answer is 'yes' (though the effective study size is small), or "does the vaccine show a result for any racial group?" in which case the answer is 'it is fairly likely we would see such a result due to chance alone.' It is worth noting how this illustrates a popular fallacy in data analysis: Frequentist hypothesis testing is not the objective exercise that some think it to be; it depends on subjective decisions about what to test.

We might decide to infer that VaxGen would have emphasized the results from any racial subgroup that showed a positive result (and the company did claim the original protocol called for analysis by racial and other subgroups [9]), and thus that they were answering the latter question. Notice that none of the options that are typically practiced or recommended are satisfying. To just report the subgroup analysis as if it were the only analyzed result obviously leads to bias. (It is worth noting that in a less high-profile research project, that might well have happened, without anyone questioning the result.) But it is not satisfying to suppress the tantalizing findings about non-whites, either because there was not really an *ex ante *hypothesis that the vaccine would work only in non-whites or because the multiple-hypothesis correction for hundreds of possible racial and other subgroups makes it non-significant. A general rule requiring us to ignore interesting but surprising findings is a huge waste of information. Requiring a data-driven subgroup analysis to be biologically plausible before reporting it offers no solution, since we can usually construct a story to explain whatever associations appear in the data (it has been speculated that some genotypes get a benefit from the vaccine, and the frequency of those genotypes is strongly correlated with race).

To offer the "hypothesis generating" caveat would make little difference, scientifically or in the securities market. It is unrealistic to suggest that this "generated" hypothesis will be re-examined given the overall disappointing result. Two studies in Thailand (one completed later [13], which also found the vaccine ineffective, and another by the U.S. National Institute of Allergy and Infectious Disease that may continue to use the vaccine anyway [14]) are likely the closest anyone will come to re-examining the hypothesis, but a population of Thais is hardly the same as non-white Americans. Furthermore, this example shows how epistemologically absurd the hypothesis generating caveat is: The result could originally have been considered hypothesis generating. But a few days after the results were released the company claimed that they had an *ex ante *plan to analyze racial and other subgroups [9], which would presumably promote the result "hypothesis confirming". However, that claim by the company, accurate or not, was completely uninformative about the effect of the vaccine, telling us nothing about the certainty of the findings, and so cannot legitimately change our conclusions. It does not matter whether the hypothesis was pre-specified. Debating whether the company really proposed the subgroup analysis *ex ante*, as if that should change our interpretation of the result, seems particularly absurd.

When Hahn et al. [3] observed apparent selective reporting of subgroup analyses, they suggested identifying subgroups in the protocol, keeping that list as short as possible, and implicitly called for reporting results for all pre-specified subgroups. But since every measured covariate creates two, several, or a continuum of possible subgroups, this approach would require ignoring a lot of the results of a study, no matter how interesting they are (as well as severely taxing the imagination of the researchers about which subgroups are the right ones). Since it is unrealistic to expect researchers to not report interesting results (let alone to not even do the analysis that would produce those results) after spending months or years gathering data, we need methods that allow the reporting of results but with less bias.

The obvious general solution is to report all subgroup analyses with equal prominence. Any reporting (be that a research paper, abstract, paper title, or press release) that suggests there is a beneficial effect for some people should equally emphasize any apparent harmful effects for other people (and vice versa). The fact that one result is statistically significant and the other is not should be of no consequence. Indeed, selecting which results to report based on statistical significance guarantees there will be publication bias (and, more generally, the inappropriate emphasis on statistical significance may be the source of a large amount of PBIS, but this point must be left for future analyses). The reporting of the multiple subgroups results should be accompanied by statistics similar to those calculated here, instead of standard test statistics, so that readers know the probability that an estimated effect (or test statistic) at least as great as the one found would result from chance alone for any of the subgroup analyses. Such information will allow readers (researchers working on related projects, policy makers, investors) to focus on what they consider to be the answer to their own questions.

## Summary

The opportunities for PBIS, along with the almost universal failure to report research results in ways that avoid it, create the possibility that biased study results are very prevalent in the health science literature. Some of the causes of PBIS are well understood, but the enormity of its implications is largely ignored.

PBIS can produce very misleading results, leading to widespread misperceptions and misguided policies. The examples presented here show just a few of the many ways that PBIS can result from random error and researchers' (usually innocent, almost always invisible, possibly quite reasonable) choices. Neither the problem nor the solution lies in the mathematics of data analysis, so answers will not be found by appealing to statistical theory. The critical issue is the completeness of reporting and the plain-language interpretation of results.

The simple solutions offered by statisticians are not satisfying or even realistic. All they really let us do is observe that in almost every research report, "the rules" have been violated. This is not helpful. Rather than a right-vs.-wrong view of proper use of statistics that would condemn most of the literature as invalid, we need a realistic way of addressing this problem. The solutions, like the solutions for traditional publication bias, will generally consist of doing a more complete job of reporting what can be reported.

## List of abbreviations

HIV = human immunodeficiency virus

OR = odds ratio

PBIS = publication bias *in situ*

## Competing interests

None declared

## Authors' contributions

Single author

## Pre-publication history

The pre-publication history for this paper can be accessed here:


